# Simultaneous Characterization and Determination of Warfarin and Its Hydroxylation Metabolites in Rat Plasma by Chiral Liquid Chromatography-Tandem Mass Spectrometry

**DOI:** 10.3390/pharmaceutics14061141

**Published:** 2022-05-27

**Authors:** Shasha Jin, Zhihong Li, Qing Yang, Boyu Fang, Xiaoqiang Xiang, Chao Peng, Weimin Cai

**Affiliations:** 1 Department of Clinical Pharmacy, School of Pharmacy, Fudan University, Shanghai 201203, China; 19111030080@fudan.edu.cn (S.J.); 19261030009@fudan.edu.cn (Q.Y.); 20211030057@fudan.edu.cn (B.F.); xiangxq@fudan.edu.cn (X.X.); 2 National Facility for Protein Science in Shanghai, Zhangjiang Lab, Shanghai Advanced Research Institute, Chinese Academy of Sciences, Shanghai 201210, China; lizhihong15@sinopharm.com

**Keywords:** enantiomers, hydroxywarfarin, LC-MS/MS, metabolites, warfarin

## Abstract

Warfarin is extensively used for venous thromboembolism and other coagulopathies. In clinical settings, warfarin is administered as a mixture of S- and R-warfarin, and both enantiomers are metabolized by multiple cytochrome P450 enzymes into many hydroxylation metabolites. Due to the high degree of structural similarity of hydroxylation metabolites, their profile possesses significant challenges. The previous methods generally suffer from lacking baseline resolution and/or involving complex analysis processes. To overcome this limitation, a sensitive and specific chiral liquid chromatography-tandem mass spectrometry (LC-MS/MS) method was developed to simultaneously identify warfarin and hydroxywarfarins enantiomers. Chromatographic separation was achieved on a HYPERSIL CHIRAL-OT column. The mass spectrometric detection was carried out in negative ion MRM mode with electrospray ionization source. The optimized method exhibited satisfactory within-run and between-run accuracy and precision with lower limit of quantification (LLOQ) of 10.0 ng/mL and 1.0 ng/mL for warfarin and 7-, 10(R)-OH-warfarin enantiomers, respectively. Linear responses of warfarin enantiomers and 7-, and 10(R)-OH-warfarin enantiomers in rat plasma were observed over the range of 10.0–8000 ng/mL, and 1.00–800 ng/mL, respectively. The analytes were shown to be stable in various experimental conditions in rat plasma. Protein precipitation was used in sample preparation without a matrix effect. This method was successfully applied to pharmacokinetic study for quantitating the concentrations of S/R-warfarin, S/R-7-OH-warfarin, and S/R-10(R)-OH-warfarin and relatively quantitating 3′-, 4-, 6-, and 8-OH warfarin enantiomers in rat plasma.

## 1. Introduction

**Figure 1:**
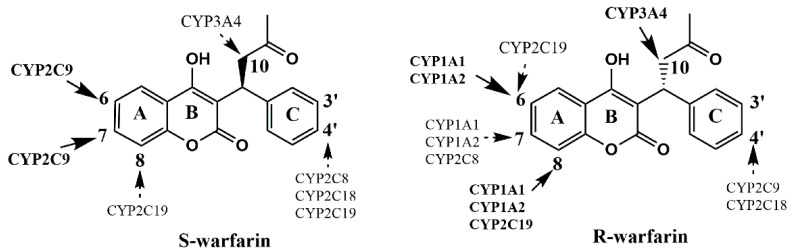
Sites of hydroxylation of R- and S-warfarin catalyzed by human P450s to form hydroxywarfarins.

Warfarin, the most commonly prescribed oral anticoagulant, is indicated for the treatment and prevention of atrial fibrillation, venous thromboembolism and other coagulopathies [1]. Warfarin is clinically administered as a mixture of the S- and R-stereoisomers [2]. S-Warfarin is a 3–5 times more potent inhibitor of the vitamin K epoxide reductase complex than R-warfarin [3]. Despite its extensive use, warfarin therapy is complicated by wide inter-individual variabilities in responses and numerous drug–drug interactions (DDIs) [4,5]. These unpredictable abilities are largely due to drug metabolism [6], as warfarin undergoes extensive hepatic metabolism into less active or inactive metabolites [7]. Warfarin is metabolized via multiple cytochrome P450 (CYP) enzymes into a series of hydroxylation metabolites by regio-selectively introducing a hydroxyl group at the different positions on the molecule (Figure 1). S-Warfarin is primarily (>80%) metabolized by CYP2C9 [2] forming S-7-hydroxy warfarin (OH-warfarin), the major metabolite observed in human plasma, and S-6-OH-warfarin [7,8]. Minor pathways involve CYP2C8, 2C18, 2C19, and CYP3A4 to form S-4′-, 8-, and 10(R;S)-OH-warfarin [9,10]. Whereas, R-warfarin is approximately 60% oxidized by CYP1A2 and 3A4, preferentially forming 10(R;S)-OH-warfarin via CYP3A4 [7]. Several other CYP enzymes, including CYP1A1, 2C18, 2C8, 2C19, and 2C9, also demonstrate minor R-4′-, 7-, 6-, and 8-hydroxylation activities [7,9,11,12,13].

Presently, it has been suggested that monitoring the individual concentrations of warfarin metabolites could identify CYP polymorphisms, leading to a better understanding of patient response to warfarin therapy [6,12]. For example, a lower concentration of S-7-OH-warfarin or R-10-OH-warfarin in plasma may indicate that the CYP2C9 or 3A4 activity is reduced, and these patients have an increased risk of bleeding events [7,8,12]. In addition, the modification of CYP2C9 and/or 3A4 mediated warfarin metabolism by co-administered drugs or food is the main reason for many clinically important DDIs [14]. Furthermore, S/R-warfarin can be recognized as a metabolic probe for a number of CYP isoforms [15]. For instance, S-warfarin has been used as the probe substrate for CYP2C9 for both in vitro and in vivo DDI studies [16]. Therefore, the profile of warfarin and its metabolites, as biological endpoints for metabolic pathways, will enable us to elucidate the dose–response relationship [6]. Furthermore, specific quantification of S-7-OH-warfarin and R-10-OH-warfarin will enable phenotypic characterization of CYP2C9 and 3A4 activities and better understanding of the mechanisms of DDIs occurring during warfarin therapy [17].

However, characterization and determination of warfarin and its hydroxylation metabolites possess significant analytical challenges due to the high degree of structural similarity of the metabolites and the great number of possible analytes. Many metabolites are isomers that differ only in regio-chemistry of a single functional group [12]. Moreover, hydroxylation at C10 creates a second chiral center resulting in four possible isomers [6], which creates even more diastereomers and enantiomeric pairs [12]. Thus, a chiral stationary phase or chiral mobile phase is necessary for analyzing the enantiomers. Currently, there are quite a few analytical methods developed for the determination of warfarin enantiomers and/or their hydroxylation metabolites in biological matrices or human plasma (Supplementary Table S1). However, only a few methods attempted to resolve all stereo- and regio-isomers of warfarin hydroxylation metabolites along with parent (R/S)-warfarin simultaneously [6,9,18,19,20]. Moreover, the previous methods generally suffer from a lack of a baseline resolution and/or involving cumbersome method development and instrumental setup. For example, a previous method failed to achieve the baseline separation for R-6, 7-, and 8-OH-warfarin [9]. In a more recent study using the method developed by Miller et al., the separation of warfarin, 4′-,6-,7-,8-, and 10-OH-warfarin enantiomers in human plasma was achieved by combining phenyl based reverse phase column and chiral column and adopting complicated sample preparation [18]. Thus, simultaneous determination of S-warfarin, R-warfarin, and their enantiomeric hydroxylation metabolites in plasma by mass spectrometric technique remains to be explored. The present study was aimed to develop a sensitive and specific LC-MS/MS method to profile warfarin and its hydroxywarfarins enantiomers.

## 2. Materials and Methods

### 2.1. Chemical and Reagents

S-(-)-warfarin (purity, 97%), R-(+)-warfarin (purity, 98%), (S)-7-hydroxy warfarin (purity, 98%), (R)-7-hydroxy warfarin (purity, 98%), (S)-10-hydroxy warfarin (purity, 97%), (R)-10-hydroxy warfarin (purity, 97%), and 3′-, 4′-, 6-, and 8-hydroxy warfarin (purity, 98%) were purchased from Toronto Research Chemicals (Toronto, ON, Canada). Warfarin sodium (purity, 98%) and diclofenac sodium (purity, ≥99%) were purchased from J&K Scientific Ltd. (Beijing, China). LC-MS grade methanol and acetonitrile were purchased from Merck (Darmstadt, Germany). Formic acid (FA) was obtained from Sigma-Aldrich (St. Louis, MO, USA). Ultra-pure water was purified by a Milli-QTM system (Millipore, Bedford, MA, USA).

### 2.2. UHPLC-MS/MS Instruments and Conditions

The analyses were performed using an Agilent 1290 Infinity Binary–ultra-high performance liquid chromatography (UHPLC) system connected to a mass spectrometer Agilent Jet Stream (AJS) electrospray ionization source Triple Quadrupole (QQQ) 6490 series (Santa Clara, CA, USA). The chromatographic separation was achieved on a HYPERSIL CHIRAL-OT column (4.6 × 150 mm, 3.0 μm, cellulose tris-(3,5-dimethylphenyl-carbamate) modified silica, ThermoFisher Scientific, Waltham, MA, USA) at 30 °C. The mobile phase consisted of 60% phase A (0.1% FA in water) and 40% phase B (0.1% FA in acetonitrile). The elution was at a flow rate of 0.40 mL/min for 50 min. The auto-sampler temperature was maintained at 10 °C with an injection volume of 2 μL. The mass spectrometer was operated in the negative mode and the final optimized ion funnel (iFunnel) parameters were: gas temperature of 250 °C; gas flow of 12 L/min; nebulizer pressure of 35 psi; sheath gas temperature of 300 °C; sheath gas flow of 11 L/min; capillary voltage of 4000 V; and nozzle voltage of 500 V. Multiple reaction monitoring (MRM) was performed to quantify warfarin enantiomers and its hydroxylation metabolites. All ionic transitions were optimized by MassHunter Optimizer and were displayed in Table 1. Agilent MassHunter Workstation^®^ (version B.06.00, Agilent Technologies Inc., Santa Clara, CA, USA) was used for system control, data acquisition, fragment optimizer, and data processing.

### 2.3. Preparation of Calibration Standards and Quality Control Samples

The stock solutions of S-(-)-warfarin, R-(+)-warfarin, (S)-7-OH-warfarin, (R)-7-OH-warfarin, (S)-10-OH-warfarin, (R)-10-OH-warfarin, and internal standard (IS) were individually prepared in methanol. The concentrations were 10.0 mg/mL for IS and 1.00 mg/mL for the others, corrected by the purity and salt correction factor of each compound. Calibration curve and quality control (QC) working solutions were prepared by serially diluting stock solutions separately in methanol–water (1:1, *v*/*v*), all of which contained the appropriate concentrations of each analyte. Stock and working solutions were stored at −20 °C.

Blank plasma was obtained from male Sprague Dawley (SD) rats by centrifuging the blood (EDTA-K2 as anticoagulant) at 3000× *g* for 10 min at 4 °C and stored at −70 °C. The calibration standards were prepared daily by spiking 90 µL of blank plasma with 10.0 µL of corresponding working solutions, which covered the concentration levels of 10.0, 20.0, 100, 500, 1000, 2000, and 8000 ng/mL for warfarin enantiomer, and 1.00, 2.00, 10.0, 50.0, 100, 200, and 800 ng/mL for 7-, 10-OH-warfarin enantiomer. The lower limit of quantitation (LLOQ), low quality control (LQC), median quality control (MQC) and high quality control (HQC) samples were prepared in the same manner at concentration levels of 10.0, 20.0, 500, and 6000 ng/mL for warfarin enantiomer, and 1.00, 2.00, 50.0, and 600 ng/mL for 7-,10-OH warfarin enantiomer, respectively.

### 2.4. Sample Preparation

The calibration standards, QC samples and plasma samples from pharmacokinetic study were pretreated simultaneously as described below. A protein precipitation procedure was performed for the sample extraction. The frozen plasma samples were thawed at room temperature and the melted samples should be fully vortexed prior to analysis. Thereafter, 100 µL plasma sample was spiked with 300 µL of methanol–acetonitrile (1:1, *v*/*v*) containing 20.0 μg/mL IS. After vortex for 5 min and centrifugation at 13,000× *g* for 10 min at 4 °C, an aliquot of 300 µL supernatant was evaporated to dryness under the nitrogen stream at room temperature. The obtained residue was reconstituted in 100 µL methanol–water (1:1, *v*/*v*), vortexed for 10 min and then centrifuged at 13,000× *g* for 10 min at 4 °C. An aliquot of 2.0 µL supernatant was injected into the UHPLC-MS/MS for analysis.

### 2.5. Method Validation

The method was validated according to the guidelines on bioanalytical method validation of the European Medicines Agency (EMA) [21]. Selectivity, lower limit of quantification (LLOQ), linearity of the calibration curve, accuracy and precision, matrix effect, carry-over, dilution integrity, and stability were evaluated. The selectivity of the method was examined by using blank plasma samples from six individual SD rats. The peak area of interference should be ˂20% of the analytes area and ≤5% of IS area in LLOQ samples. LLOQ was determined as the lowest calibration standard. The analyte signals in the LLOQ sample should be at least 5 times as much as in the blank sample and be adapted to the pharmacokinetics study. The linearity of the calibration curves was evaluated using seven non-zero calibration standard samples for each analyte prepared in three successive batches. Calibration curves were established by linear regression (weighting 1/x^2^) of the nominal analyte concentration (x) against the analyte to IS peak area (y). To assess the within- and between-run accuracy and precision, LLOQ and QC samples were conducted in three independent validation runs with six replicates. The accuracy (RR), expressed as the percentage of average concentration to the normal concentration, should be within 85–115% for the QC samples and 80–120% for the LLOQ samples. The precision, expressed as coefficient of variation (CV), should not exceed 15% for the QC samples, and 20% for the LLOQ samples. The matrix effects were evaluated by using LQC, HQC, and blank plasma samples from six-individual SD rats. For each analyte and IS, the matrix factor (MF) was estimated by calculating the ratio of the peak area in the presence of matrix (analytes spike with blank plasma) to the peak area in absence of matrix (pure analytes solution) for each lot of blank plasma. The IS normalized MF was obtained through dividing the MF of the analyte by the MF of the IS. The CV of the IS-normalized MF calculated from the six lots of blank plasma should be less than 15%. Carry-over was assessed by running a triple analysis of blank plasma sample after the upper limit of quantification (ULOQ) sample. The dilution integrity was evaluated by diluting rat plasma at the concentrations 5-fold HQCs with blank plasma using a ratio of 1:4. In this study, six replicates of LQC and HQC samples were stored at room temperature for 8 h and at −70 °C for 1 month to investigate the short term and long term stability, respectively. The autosampler stability was evaluated by storing the QC samples in the autosampler at 10 °C for 24 h. In addition, the freeze and thaw stability was assessed after the QC samples passed through three consecutive freeze and thaw cycles. For that, the QC samples were frozen at −70 °C for at least 24 h and then thawed at room temperature. Samples were considered to be stable if the mean concentrations of these QC samples were within 85–115% of the nominal concentrations.

### 2.6. Application of the Method to Pharmacokinetics of Warfarin in Rats

Sprague Dawley (SD) rats (male, 200 ± 10 g) were purchased from Sino-British Sippr/BK Lab Animal Ltd. (Shanghai, China). The animals were kept in a 12 h light–dark cycle temperature controlled room (24 ± 2 °C). Food and water were accessed freely before experiments. Rats were fasted for 12 h with free access to water before drug administration during pharmacokinetic experiments. Dosing solutions for intragastric administration were prepared by physiological saline spiked with warfarin (0.2 mg/mL). The validated UHPLC-MS/MS method was applied to quantify warfarin enantiomer and its hydroxylation metabolites to support the pharmacokinetic study after administration of warfarin (2 mg/kg). The dose of warfarin was selected according to previous literature for drug interactions in rats [22,23,24,25]. Blood samples were collected at the following time points: 0 (pre-dose), 0.5, 2, 4, 6, 8, 10, 12, 25, 32, 49, 73, and 97 h after dosing by orbital bleeding into the 1.5 mL Eppendorf tube (using EDTA-K2 as anticoagulant). Collection tubes were gently inverted for 4–5 times, and centrifuged at 3000× *g* for 5 min at 4 °C. The separated plasma samples were collected and stored at –70 °C until analysis.

### 2.7. Statistical Analysis

The mean concentration levels of warfarin and its metabolites in rat plasma versus time profile was explored by GraphPad Prism 8.0 (GraphPad Software Inc., San Diego, CA, USA), and the main pharmacokinetic parameters of warfarin and its hydroxylation metabolites were analyzed in non-compartmental mode using the Modeling and Simulation Studio (MaS Studio) software (version 1.3.0, Shanghai BioGuider Pharmaceutical Technology Co., LTD, Shanghai, China).

## 3. Results and Discussion

### 3.1. Method Development

**Figure 2:**
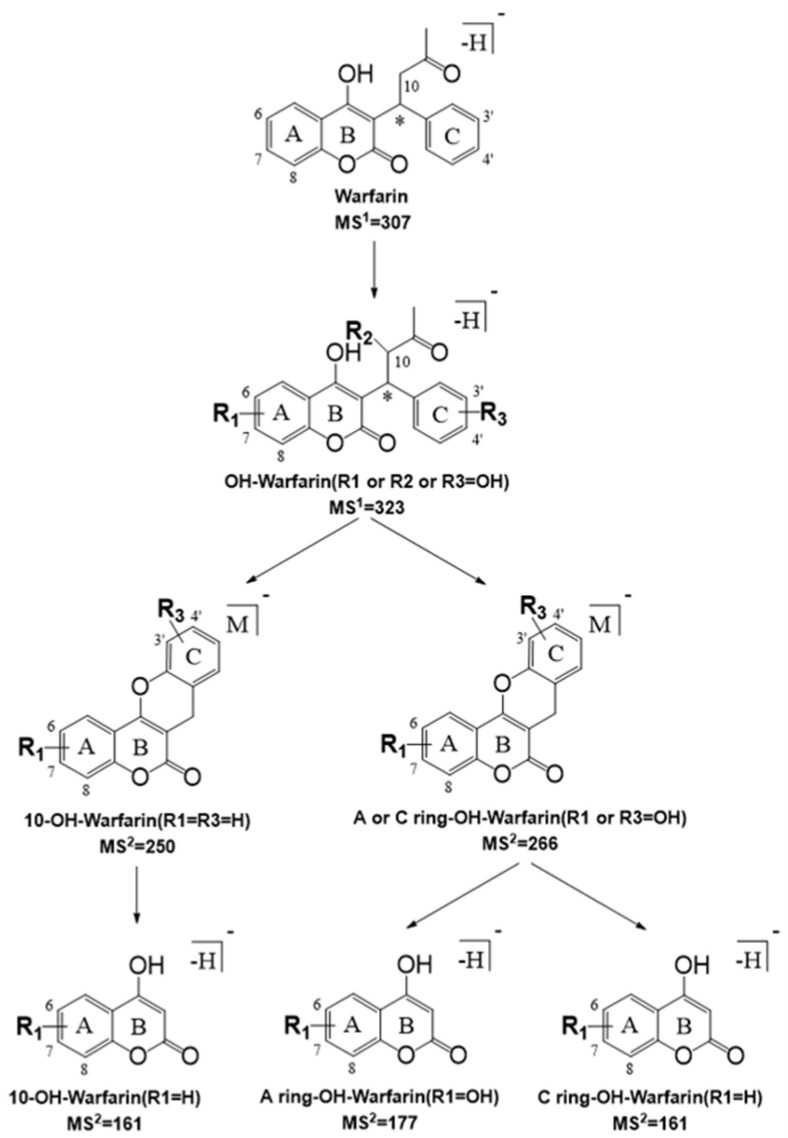
Fragment ions-based decision diagram for the identification of warfarin hydroxylation metabolites.

The major metabolic pathway of warfarin is oxidation forming various hydroxywarfarins, which comprise 80–85% of the total metabolites [7]. Warfarin hydroxylation usually occurs at three positions, namely, alkyl side chain, A-ring, and C-ring (Figure 1). Additionally, the majority of product ions of hydroxywarfarins are readily assigned due to cleavages at two of the three C-C bonds connected to the chiral C9 carbon [26,27], which provide critical information for the identification and quantification of hydroxywarfarins. Herein, a decision diagram analyzing the fragmentation pathways of hydroxywarfarins was firstly established to profile warfarin monohydroxylated metabolites in rat plasma. As demonstrated in Figure 2, 10-OH-warfarin initially produced characteristic fragment ion of *m*/*z* 250, and further produced another characteristic fragment ion of *m*/*z* 161. Both A-ring OH-warfarin and C-ring OH-warfarin could produce fragment ion of *m*/*z* 266, but the fragment ions produced by *m*/*z* 266 could distinguish A-ring OH-warfarin from C-ring OH-warfarin. The fragment ion of *m*/*z* 177 produced by *m*/*z* 266 was a special fragment ion of A-ring OH-warfarin. However, the fragment ion of *m*/*z* 161 produced by *m*/*z* 266 was a symbol for C-ring OH-warfarin. Furthermore, the targeted multi reaction monitoring (MRM) was the most common data acquisition mode of triple quadrupole mass spectrometry and had higher selectivity because analytes were detected by their special precursor ion and product ion pairs. Thus, the MRM channels (Table S2) were accordingly established to identify various warfarin hydroxylation metabolites in rat plasma according to the fragment ions mentioned above.

**Figure 3:**
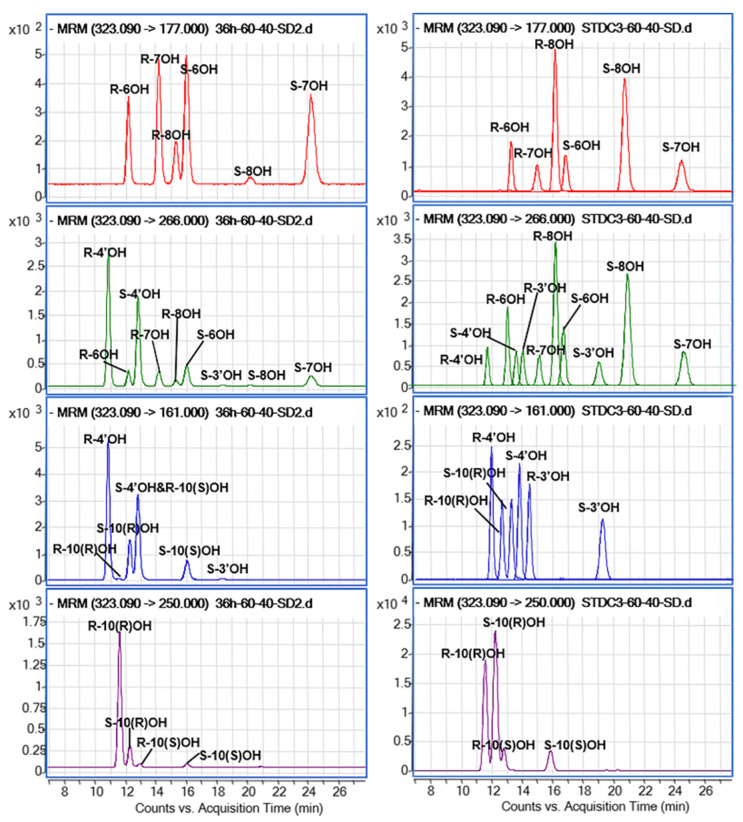
Characterization of warfarin hydroxylation metabolites in rat plasma. The targeted multiple reaction monitoring channels chromatograms of hydroxy-warfarin in plasma samples (**left** pane) and standard samples (**right** pane).

Considering the polarity and acid-base properties of warfarin and its hydroxylation metabolites, acetonitrile with 0.1% FA was used as the organic mobile phase. Several elution gradients were tested to archive the baseline separation for all hydroxywarfarin analytes. The preliminary results showed that the separation was improved by increasing the water phase ratio, resulting in a longer analysis time. Under an overall consideration of sensitivity and analysis time, the mobile phase was determined with 60% phase A (0.1% FA in water) and 40% phase B (0.1% FA in acetonitrile) at a flow rate of 0.40 mL/min for 50 min. The commercially available 3′-, 4′-, 6-, (S/R)-7-, 8-, and (S/R)-10-OH-warfarin were added as reference compounds to evaluate the validity of this approach. A total of six warfarin hydroxylated metabolites, comprising seven pairs of enantiomers, could be identified, including A-rings hydroxywarfarins (S/R-6-OH-warfarin, S/R-7-OH-warfarin, and S/R-8-OH-warfarin), C-rings hydroxywarfarins (S/R-3′-OH-warfarin and S/R-4′-OH-warfarin), and 10-hydroxywarfarins (S/R-10(R)-OH-warfarin and S/R-10(S)-OH-warfarin) (Figure 3). To our knowledge, simultaneous baseline separation of all hydroxywarfarin enantiomers has not been reported previously, although multiple groups resolved part of the warfarin and hydroxylation metabolites enantiomers [6,9,19,20]. Moreover, the testing process of elution program gave us inspiration that, theoretically, this method could be adapted to determine any target composition of warfarin and/or hydroxywarfarins enantiomers, as long as a suitable elution program and analysis time were set.

Historically, only R and S-10-hydroxywarfarin have been reported as possible metabolites [28]. However, there are four isomeric forms of 10-hydroxywarfarin, because hydroxylation at C10 creates a second chiral center [6]. The previous methods paid less to no attention to this fact. Previously, Drew R. et al. detected the four 10-hydroxywarfarin isomers by using multi-mode UPLC–MS/MS method, but could not achieve baseline separation [6]. In our method, four 10-OH-warfarin isomers were clearly shown to be separated in the rat plasma (Figure 3, left pane), although two isomers (S/R-10(S)-OH-warfarin) were below the LOQ and only S/R-10(R)-OH-warfarin were considered for the following method validation and quantitation in rat plasma. Additionally, diclofenac was chosen as the IS, as it had reasonable retention time and no interference with other analytes [29]. The specific MRM transitions of warfarin enantiomers (307→250) and diclofenac (294→250) were assigned from previously reported transitions [17,29] and optimized by MassHunter Optimizer (Table 1).

### 3.2. Method Validation

#### 3.2.1. Selectivity and Carry Over

**Figure 4:**
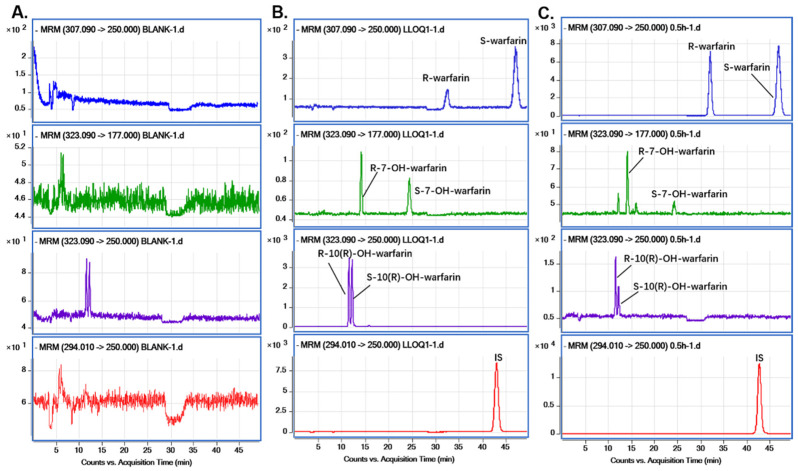
Typical multiple reaction monitoring chromatograms of blank plasma sample obtained from six-individual rats (**A**), a plasma sample of LLOQ containing S/R-warfarin, S/R-7-, 10(R)-0H-warfarin, and IS (diclofenac) (**B**), and real plasma sample collected from rat after 0.5 h oral administration of 2 mg/kg warfarin sodium (**C**).

The typical chromatograms of the blank sample (obtained from six-individual rats), LLOQ sample, and real rat plasma sample (0.5 h after administrated warfarin) were harvested and compared for potential interferences (Figure 4). The results showed that the responses of interference were <20% than the LLOQ and <5% than IS, and the accurate retention time of R-10(R)-OH-warfarin, S-10(R)-OH-warfarin, R-7-OH-warfarin, S-7-OH-warfarin, R-warfarin, and S -warfarin, were 11.618, 12.296, 14.254, 24.479, 32.398, and 47.029 min, respectively. Additionally, the carry-over in the blank plasma samples, a triple running analysis after the ULOQ samples, were less than 7.89% for all analytes and less than 0.05% for IS, which was within the acceptance criteria [21].

#### 3.2.2. Standard Curve

Standard curves were validated in the concentration range of 10.0–8000 ng/mL for warfarin enantiomers and 1.00–800 ng/mL for 7-, and 10(R)-OH-warfarin enantiomers in rat plasma. A regression analysis performed with 1/x^2^ weighting gave linear correlation over the analytical ranges. The linear correlation coefficients were over 0.99. The observed concentrations of standard samples were within 85–115% of the nominal values (*n* = 6). The lowest concentrations of the calibration range 10.0 ng/mL and 1.00 ng/mL were the LLOQs for warfarin and 7-, 10(R)-OH-warfarin enantiomers, respectively. They did not deviate by more than ±20% of the nominal values as expected by regulatory recommendations (Table S3).

#### 3.2.3. Accuracy and Precision

The within- and between-run accuracy and precision were examined by conducting three independent validation runs with six replicates for per concentration level (LLOQ, LQC, MQC, and HQC). RR and CV were calculated to demonstrate the precision and accuracy, respectively (Table 2). As required by regulatory guidelines, the within- and between-run accuracy (RR%) and precision (CV%) were within ±15% at QC levels and within ±20% at LLOQ level.

#### 3.2.4. Matrix Effect

The matrix effect of the analytes is summarized in Table 3. It can be seen that the matrix effect from rat plasma ranged between 90.71–109.40%. The CV of the IS-normalized MF calculated from the six lots of matrixes were less than 9.53%. These results indicated that there were no significant matrix effects in the present assay during the entire procedure.

#### 3.2.5. Dilution Integrity

Six replicate plasma samples with a concentration above the ULOQ were diluted five-fold with blank rat plasma. The accuracy and precision of the samples after a five-fold dilution were still within the acceptable limits for accuracy and precision (data not shown).

#### 3.2.6. Stability

The stability of the warfarin, 7-OH-warfarin, and 10(R)-OH-warfarin enantiomers was assessed in six replicates at LQC and HQC levels, under various storage and processing conditions (Table 4). These analytes’ concentrations were not affected by short storage at room temperature (8 h) or by long frozen-state storage (30 days at −70 °C). Processed samples were stable in an autosampler for 24 h (10 °C). Furthermore, three freeze/thaw cycles had no impact on these analyte concentrations after storage at −70 °C. The present stability results for warfarin, 7-OH-warfarin, and 10(R)-OH-warfarin enantiomers are in accordance with a previous study [17].

### 3.3. Quantitating Enantiomeric Warfarin and Hydroxywarfarins in Rat Plasma

**Figure 5:**
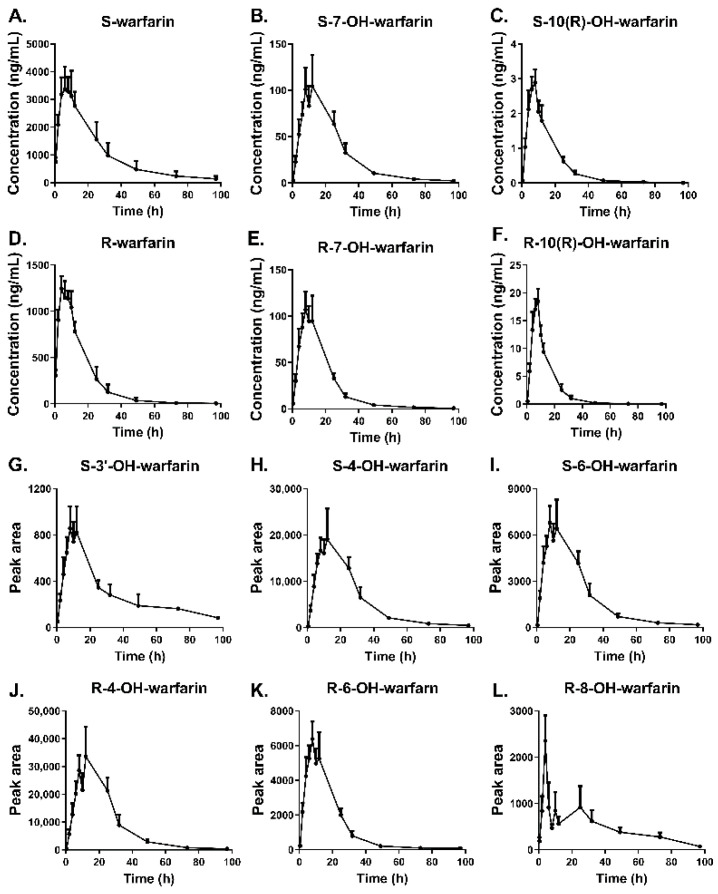
Mean plasma concentration/peak area–time curves of warfarin and its hydroxylation metabolites in SD rats after oral administration of warfarin 2 mg/kg (*n* = 8). Quantification of S/R-warfarin (**A**,**D**), S/R-7-OH-warfarin (**B**,**E**) and S/R-10(R)-OH-warfarin (**C**,**F**); and relative quantification of S-3′-OH-warfarin (**G**), S/R-4′-OH-warfarin (**H**,**J**), S/R-6-OH-warfarin (**I**,**K**), and R-8-OH-warfarin (**L**).

The enantiomeric warfarin and its six hydroxylation metabolites in rat plasma could be simultaneously identified using the presently developed chiral HPLC-MS/MS method. We applied the validated method to quantitate the concentrations of enantiomeric warfarin and 7-, 10(R)-OH-warfarin in rat plasma after oral administration warfarin (2 mg/kg). The plasma concentration–time profiles are shown in Figure 5, and their main pharmacokinetic parameters are presented in Table 5. S-warfarin was detectable up to 97 h after warfarin administration and demonstrated a much higher area under curve (AUC), maximum concentration (C_max_), half-life, and peak plasma time (T_max_) compared to R-warfarin, which is in good agreement with previous studies [30,31,32,33,34]. The discrepant profiles between S- and R-warfarin revealed the stereoselective pharmacokinetics of warfarin, that S-warfarin, the more active form, was absorbed more, metabolized less, or excreted slower than R-warfarin [34,35]. Interestingly, in several previous studies, the profiles of S- and R-warfarin were much similar with respect to AUC, C_max_, and half-life [36,37,38]. One possible explanation would be the different rat strains used in these studies. As in the present and previous studies, warfarin behaved the same stereoselective pharmacokinetics manner in both SD and Wistar rats, but presented more significantly different between S- and R-warfarin in SD rats. Furthermore, the major observed metabolites in rat plasma were S/R-7-OH-warfarin and R-10(R)-OH-warfarin, indicating the importance of CYP2C9 and 3A4 in warfarin metabolism [7]. In addition, the amount of R-10(R)-OH-warfarin (C_max_ = 20.44 ± 7.06 ng/mL) in the plasma was much higher than S-10(R)-OH-warfarin (C_max_ = 3.16 ± 1.31 ng/mL), which indicates that CYP3A4 mainly metabolizes R-enantiomer. In addition, the T_max_ of 7-OH-warfarin was later than 10-OH-warfarin, while the isomers of each metabolite have the similar T_max_.

Meanwhile, four hydroxywarfarins, 3′-, 4′-, 6-, and 8-OH-warfarin enantiomers, formed via the minor metabolic pathways in humans, were relatively quantitated in rat plasma. The curves of peak area changes of these hydroxywarfarins enantiomers during 97 h after oral administration warfarin are shown in Figure 5. The relative plasma level of R-4′-OH-warfarin was the highest, followed by S/R-6-OH-warafrin and R-8-OH-warfarin. Pohl et.al. previously reported the amount of hydroxywarfarins excreted in rat urine ordered as 4′-OH-warfarin, 6-OH-warfarin, and 8-OH-warfarin [39]. Interestingly, in humans, the 4′-hydroxylation of R-warfarin is significantly less activated by CYP2C9/2C18, and reversely the 6-hydroxylation of R-warfarin is strongly regio-selected by CYP1A2 [7]. This may indicate warfarin metabolism varied between different species, and more attention should be paid when interpreting the findings from rats to other species. Further, the peak area of S-8-OH-warfarin was small and could not be obtained from the rat plasma after 40 h administration warfarin (data not shown), which could be attributed to the stereoselective formation of 8-OH-warfarin for R-enantiomer [30]. Particularly, the S-3′-OH-warfarin in rat plasma was firstly determined in our method, which was not observed in earlier reported methods [6,9,40], although Shaik et al. could quantitate the racemic 3′-OH-warfarin by using the LC-MS/MS Qtrap [41]. Compared with previous observations, there was a common agreement on the major metabolites, and indeed some differences on the minor ones. Overall, our results clearly demonstrate that this chiral HPLC–MS/MS method is suitable for enantiomeric profile warfarin enantiomers and its hydroxylation metabolites in rat plasma. Prospectively, with requisite validation, this method could be used to analyze warfarin metabolism samples derived from other in vitro or in vivo sources (e.g., liver microsomes incubation mixture or human plasma).

## 4. Conclusions

A simple and sensitive chiral HPLC–MS/MS analytical method was developed for simultaneous characterization and determination of warfarin enantiomers and its six enantiomeric hydroxylation metabolites. This method has been validated with satisfactory accuracy and adequate reproducibility for quantitating warfarin, 7-, and 10(R)-OH-warfarin enantiomers in rat plasma. This method has the advantages of easy sample preparation, simple method development and instrumental setup, and distinct baseline resolution. It provides a valuable tool to effectively assess the widest array of warfarin metabolites, leading to a better understanding of warfarin metabolism and interindividual differences to responses during warfarin therapy. Moreover, due to its simplicity and reproducibility, it could be easily applied to other types of biological matrices for preclinical or clinical use after necessary validations.

## Figures and Tables

**Table 1:** Mass spectrometric parameters of the individual analytes.

Analytes	Precursor Ion (Da)	Product Ion (Da)	FM (V)	CE (V)	CAV (V)
S/R-warfarin	307.09	250	250	25	3
S/R-6-OH-warfarin	323.09	177	250	21	3
S/R-7-OH-warfarin	323.09	177	250	21	3
S/R-8-OH-warfarin	323.09	177	250	21	3
S/R-3′-OH-warfarin	323.09	266	250	25	3
S/R-4′-OH-warfarin	323.09	266	250	25	3
S/R-10(R;S)-OH-warfarin	232.09	250	250	21	3
Diclofenac (IS)	294.01	250	250	9	3

CAV, cell accelerator voltage; CE, collision energy; FM, fragmentor; and IS, internal standard.

**Table 2:** The within- and between-run accuracy and precision of warfarin and its hydroxylation metabolites in rat plasma.

Analytes		Within-Run		Between-Run	
	Levels (ng/mL)	Accuracy (RR, %)	Precision (CV, %)	Accuracy (RR, %)	Precision (CV, %)
R-10(R)-OH-warfarin	1	93.28	6.90	98.12	12.57
	2	95.22	12.18	96.58	7.51
	50	101.50	4.15	101.83	3.69
	600	100.37	5.08	98.23	6.22
S-10(R)-OH-warfarin	1	100.18	12.92	99.26	11.86
	2	109.28	9.54	103.78	8.47
	50	104.54	5.97	103.88	4.08
	600	101.89	3.80	102.04	3.42
R-7-OH- warfarin	1	97.52	6.57	103.71	9.95
	2	93.16	5.55	98.89	7.77
	50	103.17	8.03	99.48	7.51
	600	101.37	4.35	104.45	6.14
S-7-OH- warfarin	1	110.22	6.70	109.28	15.82
	2	108.25	8.47	102.10	8.06
	50	108.42	3.97	102.55	8.55
	600	101.65	3.73	101.62	8.72
R-warfarin	10	111.85	9.91	105.41	10.33
	20	107.34	7.04	103.89	11.20
	500	102.54	11.18	104.20	9.42
	6000	92.80	13.29	97.37	9.62
S-warfarin	10	107.26	15.48	106.43	10.98
	20	106.85	12.35	101.88	10.23
	500	100.78	3.92	97.42	9.89
	6000	101.13	8.45	98.89	6.58

CV, coefficient of variation; RR, relative recovery.

**Table 3:** Matrix effect of warfarin and hydroxywarfarins enantiomers in rat plasma.

Analytes	Levels (ng/mL)	MF/MF_i_ (*n* = 6)	
		Mean (%)	CV (%)
R-10(R)-OH-warfarin	2	98.35	5.01
	600	96.97	7.18
S-10(R)-OH-warfarin	2	92.33	5.19
	600	100.95	9.53
R-7-OH-warfarin	2	96.28	7.31
	600	97.04	7.54
S-7-OH-warfarin	2	98.48	8.33
	600	98.83	5.73
R-warfarin	20	95.50	5.96
	6000	90.71	8.58
S-warfarin	20	109.40	4.63
	6000	94.11	5.35

MF, matrix effect; MF_i_, IS normalized MF.

**Table 4:** Stability results of warfarin and hydroxywarfarins enantiomers in rat plasma under different conditions.

Conditions	Levels (ng/mL)	Autosampler for 24 h (*n* = 6)		Room Temperature for 8 h (*n* = 6)		Three Treeze-Thaw Cycles (*n* = 6)		–70 °C for 30 Days (*n* = 6)	
		RR (%)	CV (%)	RR (%)	CV (%)	RR (%)	CV (%)	RR (%)	CV (%)
R-10(R)-OH-warfarin	2	95.39	4.72	99.11	3.55	103.79	8.30	98.40	7.59
	600	100.54	6.12	91.58	1.25	104.35	6.67	102.11	7.06
S-10(R)-OH-warfarin	2	103.93	5.40	98.12	7.05	94.89	9.15	96.43	5.76
	600	102.06	3.97	102.23	2.84	101.28	4.40	98.04	5.98
R-7-OH- warfarin	2	107.75	4.04	95.76	3.25	110.48	5.79	107.27	2.47
	600	106.83	5.98	90.41	8.14	103.49	3.39	101.79	5.20
S-7-OH- warfarin	2	94.13	2.38	103.91	3.81	103.36	7.14	101.46	9.92
	600	103.37	6.40	98.94	16.25	103.39	6.57	100.22	2.55
R-warfarin	20	100.99	15.7	103.34	10.87	100.52	11.26	94.53	6.01
	6000	104.10	4.32	94.12	2.38	100.28	10.60	97.23	10.63
S-warfarin	20	100.59	5.88	98.19	10.79	94.13	12.61	91.99	8.42
	6000	94.69	2.54	101.83	4.73	98.50	4.24	99.77	4.72

CV, coefficient of variation; RR, relative recovery.

**Table 5:** Pharmacokinetic parameters of warfarin and its main hydroxylation metabolites after oral administration of warfarin (2 mg/kg) to SD rats (*n* = 8, Mean ± SD).

Compound	AUC_0–t_ (ng·h/mL)	AUC_0–∞_ (ng·h/mL)	t_1/2_ (h)	T_max_ (h)	C_max_ (ng/mL)
S-warfarin	85,828.22 ± 69,020.95	104,043.2 ± 79,082.4	18.19 ± 3.17	8.75 ± 2.60	3371.8 ± 1737.7
R-warfarin	20,903.0 ± 10,207.9	22,567.8 ± 10,272.9	6.72 ± 3.06	4.50 ± 2.07	1223.1 ± 336.3
S-7-OH-warfarin	2755.6 ± 1726.6	2834.0 ± 1702.34	19.41 ± 14.03	10.25 ± 1.67	128.63 ± 80.71
R-7-OH-warfarin	2057.50 ± 1132.64	2079.01 ± 1129.48	12.64 ± 3.81	9.25 ± 1.83	130.67 ± 59.96
R-10(R)-OH-warfarin	240.49 ± 96.73	243.26 ± 98.92	6.50 ± 3.49	7.25 ± 2.12	20.44 ± 7.07
S-10(R)-OH-warfarin	45.82 ± 29.55	51.28 ± 28.78	10.77 ± 5.15	7.00 ± 2.14	3.16 ± 1.31

AUC_0–∞_, area under concentration-time curve from time 0 to infinity; AUC_0–t_, area under concentration-time curve from time 0 to the last time; C_max_, maximum concentration; t_1/2_: elimination half-life; and T_max_, peak plasma time.

## Supplementary Materials

The following supporting information can be downloaded at: https://www.mdpi.com/article/10.3390/pharmaceutics14061141/s1, Table S1: Overview of previously published bioanalytical methods that quantify enantiomeric warfarin and hydroxywarfarins [6,9,17,19,20,27,28,29,40,41,42,43]; Table S2: The targeted MRM channels for hydroxywarfarins; Table S3: The calibration curves, linearity range, and LLOQs of the assay (*n* = 6).
