# Supplementary material for: Simultaneous Characterization and Determination of Warfarin and Its Hydroxylation Metabolites in Rat Plasma by Chiral Liquid Chromatography-Tandem Mass Spectrometry
Source: Pharmaceutics. 2022 May 27;14(6):1141. doi: 10.3390/pharmaceutics14061141 (PMC9228315; doi:10.3390/pharmaceutics14061141)
Supplement: Supplementary file 1 [file pharmaceutics-14-01141-s001.zip › pharmaceutics-1715440-supplementary.pdf]

Supplementary Materials: Simultaneous characterization and determination of warfarin and its hydroxylation metabolites in rat plasma by chiral liquid chromatography-tandem mass spectrometry

Shasha Jin[1,#], Zhihong Li[2,#], Qing Yang[1], Boyu Fang[1], Xiaoqiang Xiang[1], Chao Peng[2,*], Weimin Cai[1,*]

[1] Department of Clinical Pharmacy, School of Pharmacy, Fudan University, Shanghai, 201203, China.

[2] National Facility for Protein Science in Shanghai, Zhangjiang Lab, Shanghai Advanced Research Institute, Chinese Academy of Science, Shanghai, 201210, China.

* Correspondence

Weimin Cai, Fudan University, 826 Zhangheng Road, Shanghai 201203, China. Email: weimincai@fudan.edu.cn

Chao Peng, National Facility for Protein Science in Shanghai, 333 Haike Road, Shanghai 201210, China. Email: pengchao@sari.ac.cn

[#] These authors contributed equally to this work.

Table S1. Overview of previously published bioanalytical methods that quantify enantiomeric warfarin and hydroxywarfarins.

Analytical method	Analytes quantified	Extraction	Samples	Analysis time	Quantification range	LOQ	References
LC-MS/MS	WAR,<br>6-, 7-, 8-, 10-OH-WAR	PP	HLM incubation mixture (250μL)	10min	2-1000 nM;<br>2 -5000 nM	2nM	Zhang et al., 2001 [27]
HPLC-MS	WAR,<br>4′-, 6-, 7-,8-, 10-OH-WAR	SPE	human plasma(100μL) and urine (50μL)	13min	0.04–10μM;<br>2.5–500 nM	2.5 nM	Ufer et al. 2004 [42]
HPLC-UV	WAR enantiomers,<br>4′-,6-,7-,8-,10-OH-WAR	LLE	human plasma (1mL)	18min	75–2500ng/mL	25 ng/ml	Locatelli, I. et al., 2005 [40]
LC-MS/MS	WAR enantiomers, S-4′-,6-,7-,8-WAR, S/R-10-OH-WAR	PP	HLM incubation mixture	30min	0.5-100 nM	2-9nM	Miller et al., 2009 [9]
LC-MS/MS	WAR,<br>4′-, 6-,7-,8-,10-OH-WAR	PP	HLM incubation mixture	5min	-	2ng/mL	Jones et al., 2010 [28]
LC–MS/MS	WAR enantiomers,<br>S/R-7-OH-WAR	SPE	human plasma (200μL)	17 min	5–1500 ng/mL;<br>75–180 ng/mL	1.5ng/ml;<br>5ng/mL	Zuo et al., 2010 [29]
UPLC–MS/MS	WAR enantiomers,<br>S/R-4′-, 6-, 7-, 8-, 10- OH-WAR	PP; SPE; LLE	HLM incubation mixture and human plasma (50μL)	17min	6.25-1000 nM	10 nM	Jones et al., 2011 [6]
MECK-MS	WAR enantiomers,<br>S/R-4′-,6-,7-, 8-, 10-OH-WAR	MAX	Human plasma (250μL)	45min	2–5000 ng/mL;<br>5–1000 ng/mL;<br>10–1000 ng/mL	0.5ng/mL;<br>1.5 ng/mL	Wang et al., 2013 [20]
HPLC-MS/MS	WAR enantiomers,<br>S/R-7-, 10-OH-WAR	PP	Human plasma (50μL)	6min	0.25–5000 nM;<br>0.1–1000 nM	1.25 nM;<br>0.5 nM	Wu et al., 2014 [17]

2D LC/Q-TOF	WAR enantiomers, S/R-4′-,6-,7-,8-,10-OH-WAR	PP	rat microsomal and plated hepatocyte incubations mixture	25min	1-1000 mg/mL; 2.5-1000ng/mL; 5-2500ng/mL	1-5 ng/mL	Joseph et al., 2015 [19]
LC-MS/MS QTrap	WAR enantiomers, 3′-,4′-,6-,7-,8-,10-OH-WAR	PP	HLM incubation mixture (50μL)	22min	1–2000 nM	1-3 nM	Shaik et al., 2016 [41]
LC–MS/MS DMS	WAR, 3′-,4′-,6-,7-,8-,10-OH-WAR	PP	HLM incubation mixture (50μL)	2.8min	100-2000nM	100 nM	Shaik et al., 2016 [41]
LC–MS/MS	WAR, 7-OH-WAR	PP	human plasma (50μL)	3min	5-2000ng/mL	5ng	Shakleya, D., et al., 2019 [43]

DMS, differential mobility spectrometry; HLM, human liver microsomes; LC-MS/MS, liquid chromatography-tandem mass spectrometry; LLE, liquid-liquid extraction; LOQ, The limit of quantitation; MAX, Mixed mode anion-exchange; MECK-MS, Micellar electrokinetic chromatography-tandem mass spectrometry; PP, protein precipitation; SPE, solid-phase extraction; WAR, warfarin.

Table S2. The targeted MRM channels for hydroxywarfarins.

Hydroxylation positions	Representative hydroxywarfarins	MRM channels
Alkyl side chain	10-OH-warfarin	323→250; 323→161
A-ring	6-OH-warfarin<br>7-OH-warfarin<br>8-OH-warfarin	323→266; 323→177
C-ring	3'-OH-warfarin<br>4'-OH-warfarin	323→266; 323→161

Table S3. The calibration curves, linearity range, and LLOQs of the assay (n=6).

Analytes	Regression equation	r^2	Weight	Linearity range (ng/mL)	LLOQs (ng/mL)	Accuracy (RR, %)
R-10(R)-OH-warfarin	y=48.105123*x+0.076899	0.9992	$1/x^2$	1.00–800	1.00	101.33
S-10(R)-OH-warfarin	y=53.071589*x+0.068172	0.9968	$1/x^2$	1.00–800	1.00	106.81
R-7-OH-warfarin	y=4.804920*x-2.362246E-004	0.9952	$1/x^2$	1.00–800	1.00	98.38
S-7-OH-warfarin	y=5.835653*x-5.631782E-004	0.9914	$1/x^2$	1.00–800	1.00	98.94
R-warfarin	y=2.019342*x-0.003800	0.9988	$1/x^2$	10.0–8000	10.0	103.76
S-warfarin	y=13.309309*x-0.003935	0.9955	$1/x^2$	10.0–8000	10.0	101.15

CV, coefficient of variation; LLOQs, the lower limit of quantitation; RR, relative recovery.
